# Medical Text Simplification Using Reinforcement Learning (TESLEA): Deep Learning–Based Text Simplification Approach

**DOI:** 10.2196/38095

**Published:** 2022-11-18

**Authors:** Atharva Phatak, David W Savage, Robert Ohle, Jonathan Smith, Vijay Mago

**Affiliations:** 1 Department of Computer Science Lakehead University Thunder Bay, ON Canada; 2 NOSM University Thunder Bay, ON Canada; 3 NOSM University Sudbury, ON Canada

**Keywords:** medical text simplification, reinforcement learning, natural language processing, manual evaluation

## Abstract

**Background:**

In most cases, the abstracts of articles in the medical domain are publicly available. Although these are accessible by everyone, they are hard to comprehend for a wider audience due to the complex medical vocabulary. Thus, simplifying these complex abstracts is essential to make medical research accessible to the general public.

**Objective:**

This study aims to develop a deep learning–based text simplification (TS) approach that converts complex medical text into a simpler version while maintaining the quality of the generated text.

**Methods:**

A TS approach using reinforcement learning and transformer–based language models was developed. Relevance reward, Flesch-Kincaid reward, and lexical simplicity reward were optimized to help simplify jargon-dense complex medical paragraphs to their simpler versions while retaining the quality of the text. The model was trained using 3568 complex-simple medical paragraphs and evaluated on 480 paragraphs via the help of automated metrics and human annotation.

**Results:**

The proposed method outperformed previous baselines on Flesch-Kincaid scores (11.84) and achieved comparable performance with other baselines when measured using ROUGE-1 (0.39), ROUGE-2 (0.11), and SARI scores (0.40). Manual evaluation showed that percentage agreement between human annotators was more than 70% when factors such as fluency, coherence, and adequacy were considered.

**Conclusions:**

A unique medical TS approach is successfully developed that leverages reinforcement learning and accurately simplifies complex medical paragraphs, thereby increasing their readability. The proposed TS approach can be applied to automatically generate simplified text for complex medical text data, which would enhance the accessibility of biomedical research to a wider audience.

## Introduction

### Background

Research from the field of biomedicine contains essential information about new clinical trials on topics related to new drugs and treatments for a variety of diseases. Although this information is publicly available, it often has complex medical terminology, making it difficult for the general public to understand. One way to address this problem is by converting the complex medical text into a simpler language that can be understood by a wider audience. Although manual text simplification (TS) is one way to address the problem, it cannot be scaled to the rapidly expanding body of biomedical literature. Therefore, there is a need for the development of *natural language processing* approaches that can automatically perform TS.

### Related Studies

#### TS Approaches

Initial research in the field of TS focused on *lexical simplification* (LS) [1,2]. An LS system typically involves replacing complex words with their simpler alternatives using lexical databases, such as the *Paraphrase Database* [3], WordNet [4], or using language models, such as *bidirectional encoder representations from transformer*s (BERT) [5]. Recent research defines TS as a *sequence-to-sequence* (seq2seq) task and has approached it by leveraging model architectures from other seq2seq tasks such as machine translation and text summarization [6-8]. Nisioi et al [9] proposed a neural *seq2seq* model, which used *long short-term memories* (LSTMs) for automatic TS. It was trained on simple-complex sentence pairs and showed through human evaluations that the TS system–generated outputs ultimately preserved meaning and were grammatically correct [9]. Afzal et al [10] incorporated LSTMs to create a quality-aware text summarization system for medical data. Zhang and Lapata [11] developed an LSTM-based neural encoder-decoder TS model and trained it using *reinforcement learning* (RL) to directly optimize SARI [12] scores along with a few other rewards. SARI is a widely used metric for automatic evaluation of TS.

With the recent progress in natural language processing research, LSTM-based models were outperformed by transformer [13]-based language models [13-16]. Transformers follow an encoder-decoder structure with both the encoder and decoder made up of *L* identical layers. Each layer consists of 2 sublayers, one being a feed-forward layer and the other a multihead attention layer. Transformer-based language models, such as BART [14], generative pretraining transformer (GPT) [15], and *text-to-text-transfer-transformer* [16], have achieved strong performance on natural language generation tasks such as text summarization and machine translation.

Building on the success of transformer-based language models, recently Martin et al [17] introduced *multilingual unsupervised sentence simplification* (MUSS) [17], a BART [14]-based language model, which achieved state-of-the-art performance on TS benchmarks by training on paraphrases mined from CCNet [18] corpus. Zhao et al [19] proposed a semisupervised approach that incorporated the back-translation architecture along with denoising autoencoders for the purpose of automatic TS. Unsupervised TS is also an active area of research but has been primarily limited to LS. However, in a recent study, Surya et al [20] proposed an unsupervised approach to perform TS at both the lexical and syntactic levels. In general, research in the field of TS has been focused mostly on sentence-level simplification. However, Sun et al [21] proposed a document-level data set (D-wikipedia) and baseline models to perform document-level simplification. Similarly, Devaraj et al [8] proposed a BART [14]-based model that was trained using unlikelihood loss for the purpose of paragraph-level medical TS. Although their training regime penalizes the terms considered “jargon” and increases the readability, the generated text has lower quality and diversity [8]. Thus, the lack of document- or paragraph-level simplification makes this an important work in the advancement of the field.

#### TS Data Sets

The majority of TS research uses data extracted from Wikipedia and news articles [11,22,23]. These data sets are paired sentence-level data sets (ie, for each complex sentence, there is a corresponding simple sentence). TS systems have heavily relied on sentence-level data sets, extracted from regular and simple English Wikipedia, such as WikiLarge [11], because they are publicly available. It was later shown by Xu [24] that there are issues with data quality for the data sets extracted from Wikipedia. They proposed the Newsela corpus, which was created by educators who rewrote news articles for different school-grade levels. Automatic sentence alignment methods [25] were used on the Newsela corpus to create a sentence-level TS data set. Despite the advancements in research on sentence-level simplification, there is a need for TS systems that can simplify text at a paragraph level.

**Figure 1:**
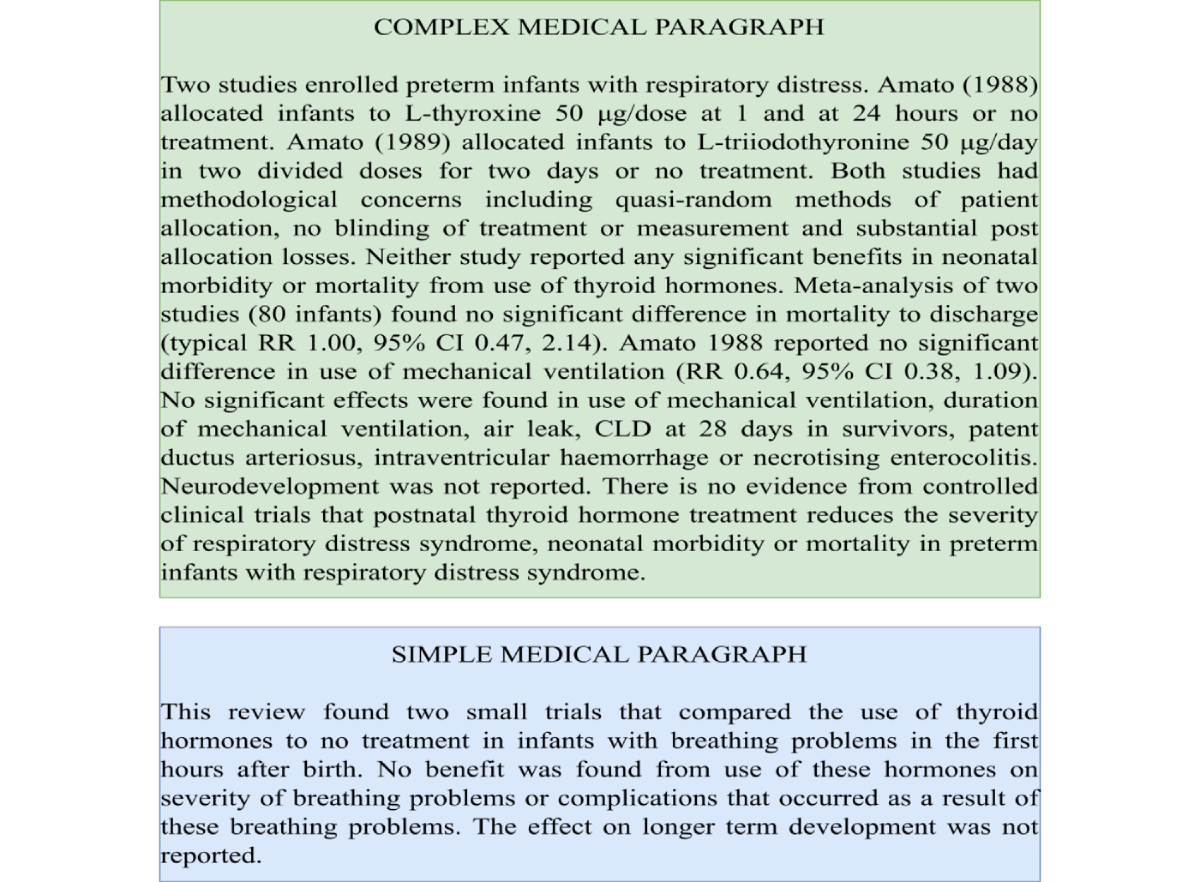
Complex medical paragraph and the corresponding simple medical paragraph from the dataset.

Recent work has focused on the construction of document-level simplification data sets [17,21,26]. Sun et al [21] constructed a document-level data set, called D-Wikipedia, by aligning the English Wikipedia and Simple English Wikipedia spanning 143,546 article pairs. Although there are many data sets available for sentence-level TS, data sets for domain-specific paragraph-level TS are lacking. In the field of medical TS, Van den Bercken et al [27] constructed a sentence-level simplification data set using sentence alignment methods. Recently, Devaraj et al [8] proposed the first paragraph-level medical simplification data set, containing 4459 simple-complex pairs of text, and this is the data set used for the analysis and baseline training in this study. A snippet of a complex paragraph and its simplified version from the data set proposed by Devaraj et al [8] is shown in Figure 1. The data set is open sourced and publicly available [28].

#### TS Evaluation

The evaluation of TS usually falls into 2 categories: automatic evaluations and manual (ie, human) evaluations. Because of the subjective nature of TS, it has been suggested that the best approach is to perform manual evaluations, based on criteria such as fluency, meaning preservation, and simplicity [20]. Automatic evaluation metrics most commonly used include readability indices such as Flesch-Kincaid Reading Ease [29], *Flesch-Kincaid Grade Level* (FKGL) [29], *Automated Readability Index* (ARI), Coleman-Liau index, and metrics for natural language generation tasks such as SARI [12] and BLEU [30].

Readability indices are used to assign a grade level to text signifying its simplicity. All the readability indices are calculated using some combination of word weighting, syllable, letter, or word counts, and are shown to measure some level of simplicity. Automatic evaluation metrics, such as BLEU [30] and SARI [12], are widely used in TS research, with SARI [12] having specifically been developed for TS tasks. SARI is computed by comparing the generated simplifications with both the source and target references. It computes an average of *F*_1_-score for 3 *n-gram* overlap operations: additions, keeps, and deletions. Both BLEU [30] and SARI [12] are n-gram–based metrics, which may fail to capture the semantics of the generated text.

### Objective

The aim of this study is to develop an automatic TS approach that is capable of simplifying medical text data at a paragraph level, with the goal of providing greater accessibility of biomedical research. This paper uses RL-based training to directly optimize 2 properties of simplified text: relevance and simplicity. *Relevance* is defined as simplified text that retains salient and semantic information from the original article. *Simplicity* is defined as simplified text that is easy to understand and lexically simple. These 2 properties are optimized using TS-specific rewards, resulting in a system that outperforms previous baselines on Flesch-Kincaid scores. Extensive human evaluations are conducted with the help of domain experts to judge the quality of the generated text.

The remainder of the paper is organized as follows: The “Methods” section provides details on the data set, the training procedure, and the proposed model, and describes how automatic and human evaluations were conducted to analyze the outputs generated by the proposed model (TESLEA). The “Results” section provides a brief description of the baseline models and the results obtained by conducting automatic and manual evaluation of the generated text. Finally under the “Discussion” section, we highlight the limitations, future work, and draw conclusions.

## Methods

### Model Objective

Given a complex medical paragraph, the goal of this work is to generate a simplified paragraph that is concise and captures the salient information expressed in the complex text. To accomplish this, an RL-based simplification model is proposed, which optimizes multiple rewards during training, and is tuned using a paragraph-level medical TS data set.

### Data Set

The Cochrane Database of Scientific Reviews is a health care database with information on a wide range of clinical topics. Each review includes a plain language summary (PLS) written by the authors who follow guidelines to structure the summaries. PLSs are supposed to be clear, understandable, and accessible, especially for a general audience not familiar with the field of medicine. PLSs are highly heterogeneous in nature, and are not paired (ie, for every complex sentence there may not be a corresponding simpler version). However, Devaraj et al [8] used the Cochrane Database of Scientific Reviews data to produce a paired data set, which has 4459 pairs of complex-simple text, with each text containing less than 1024 tokens so that it can be fed into the BART [14] model for the purpose of TS. The pioneering data set developed by Devaraj et al [8] is used in this study for training the models and is publicly available [28].

### TESLEA: TS Using RL

#### Model and Rewards

The TS solution proposed for the task of simplifying complex medical text uses an RL-based simplification model, which optimizes multiple rewards (*relevance reward*, *Flesch-Kincaid Grade rewards, and lexical simplicity rewards*) to achieve a more complete and concise simplification*.* The following subsections introduce the computation of these rewards, along with the training procedure.

#### Relevance Reward

Relevance reward measures how well the semantics of the target text is captured in its simplified version. This is calculated by computing the cosine similarity between the target text embedding (*E_T_*) and the generated text embedding (*E_G_*). BioSentVec [31], a text embedding model trained on medical documents, is used to generate the text embeddings. The steps to calculate the relevance score are depicted in Algorithm 1.


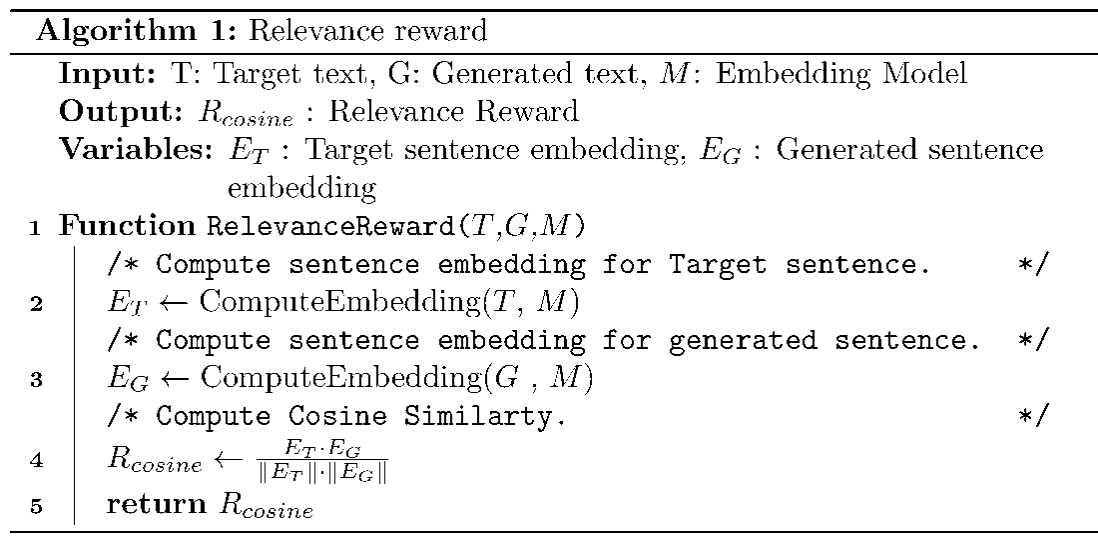


The *RelevanceReward* function takes 3 arguments as input, namely, target text (*T*), generated text (*G*), and the embedding model (*M*). The function *ComputeEmbedding* takes the input text and embedding model (*M*) as input and generates the relevant text embedding. Finally, cosine similarity between generated text embedding (*E_G_*) and target text embedding (*E_T_*) is calculated to get the reward (Algorithm 1, line 4).

#### Flesch-Kincaid Grade Reward

FKGL refers to the grade level that must be attained to comprehend the presented information. A higher FKGL score indicates that the text is more complex, and a lower score indicates that the text is simpler. The FKGL for a text (S) is calculated using equation 1 [29]:

FKGL(S) = 0.38 × (total words/total sentences) + 1.8 × (total syllables/total words) – (15.59) **(1)**

The FKGL reward (*R_Flesch_*) is designed to reduce the complexity of generated text and is calculated as presented in Algorithm 2.


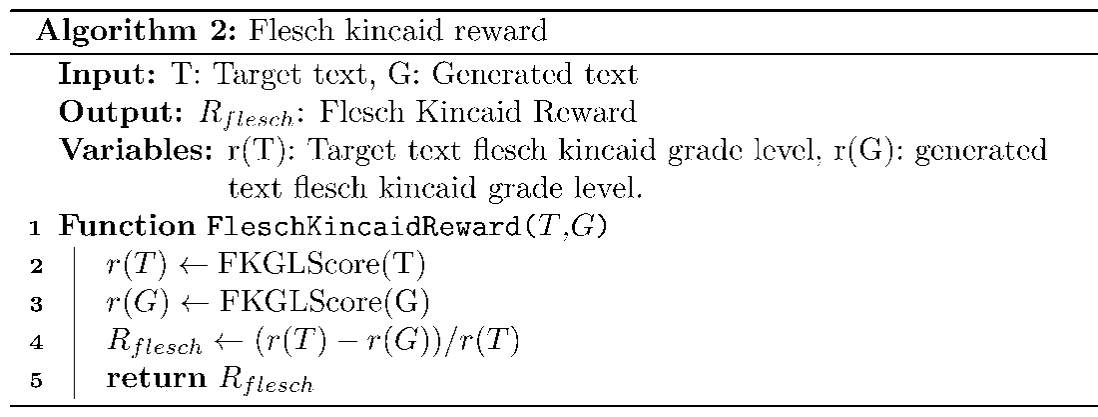


In Algorithm 2, the function *FleschKincaidReward* takes 2 arguments as inputs, namely, generated text (*G*) and target text (*T*). The *FKGLScore* function calculates the FKGL for the given text. Once the FKGL for *T* and *G* is calculated, the Flesch-Kincaid reward (*R_Flesch_*) is calculated as the relative difference between *r*(*T*) and *r*(*G*) (Algorithm 2, line 4), where *r*(*T*) and *r*(*G*) denote the FKGL of the target and generated text.

#### Lexical Simplicity Reward

Lexical simplicity is used to measure whether the words in the generated text (*G*) are simpler than the words in the source text (*S*). Laban et al [26] proposed a lexical simplicity reward that uses the correlation between word difficulty and word frequency [32]. As word frequency follows *zipf law*, Laban et al [26] used it to design the reward function, which involves calculating *zipf* frequency of newly inserted words, that is, *Z*(*G* – *S*), and deleted words, that is, *Z*(*S – G*). The lexical simplicity reward is defined in the same way as proposed by Laban et al [26] and is described in Algorithm 3. The analysis of the data set proposed by Devaraj et al [8] revealed that 87% of simple and complex pairs have a value of Δ*Z*(*S*, *G*) ≈ 0.4, where Δ*Z*(*S*, *G*) = *Z*(*G* – *S*) – *Z*(*S* – *G*) is the difference between the *zipf* frequency of inserted words and deleted words, with the value of lexical reward (*R_lexical_*) scaled between 0 and 1.

In Algorithm 3, *LexicalSimplicityReward* requires the source text (*S*) and the generated text (*G*) as the inputs. Functions *ZIPFInserted* [25] and *ZIPFDeleted* [25] calculate the *zipf* frequency of newly inserted words and the deleted words. Finally, the lexical reward (*R_lexical_*) is calculated and normalized, as described in line 5.


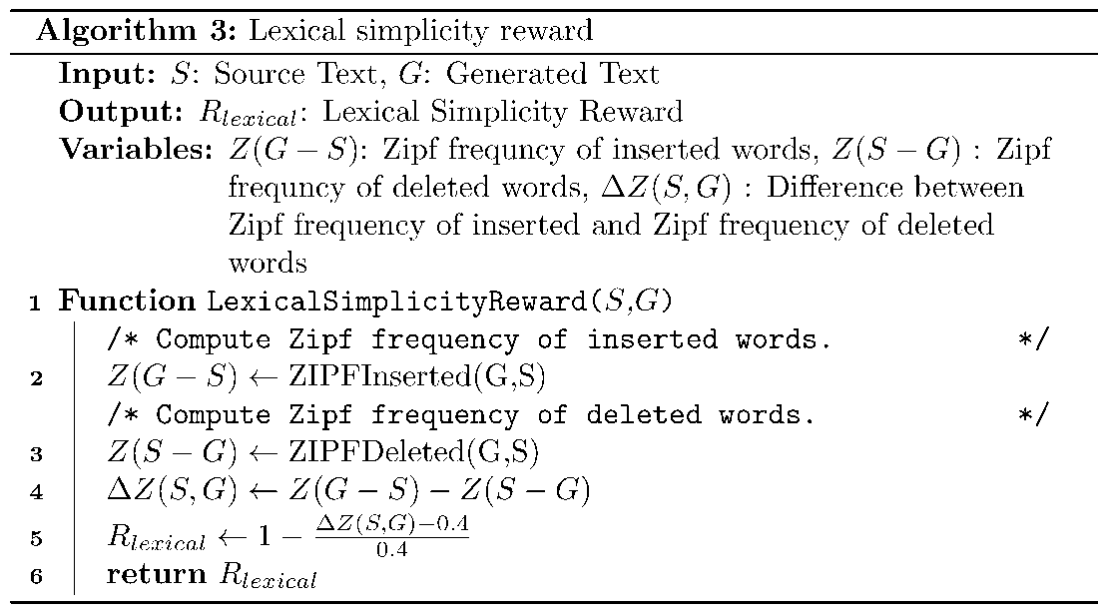


### Training Procedure and Baseline Model

#### Pretrained BART

The baseline language model used in this study for performing simplification was BART [14], which is a transformer based encoder-decoder model that was pretrained using a denoising objective function. The decoder part of the model is autoregressive in nature, making it more suitable for sentence-generation tasks. Furthermore, the BART model achieves strong performance on natural language generation tasks such as summarization, which was demonstrated on XSum [33] and CNN/Daily Mail [34] data sets. In this case, a version of BART fine-tuned on XSUM [33] data set is being used.

#### Language Model Fine-tuning

Transformer-based language models are pretrained on a large corpus of text and later fine-tuned on a downstream task by minimizing the maximum likelihood loss (*Lml*) function [3]. Consider a paired data set *C*, where each instance consists of a source sentence containing *n* tokens *x* = {*x*_1_,…,*x_n_*} and target sequence containing *m* tokens *y* = {*y*_1_,…,*y_n_*}, then the *Lml* function is given in equation 2 with the computation described in Algorithm 4.



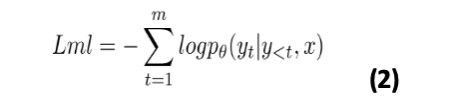



where *θ* represents the model parameters and *y*_<_*_t_* denotes preceding tokens before the position *t* [35].


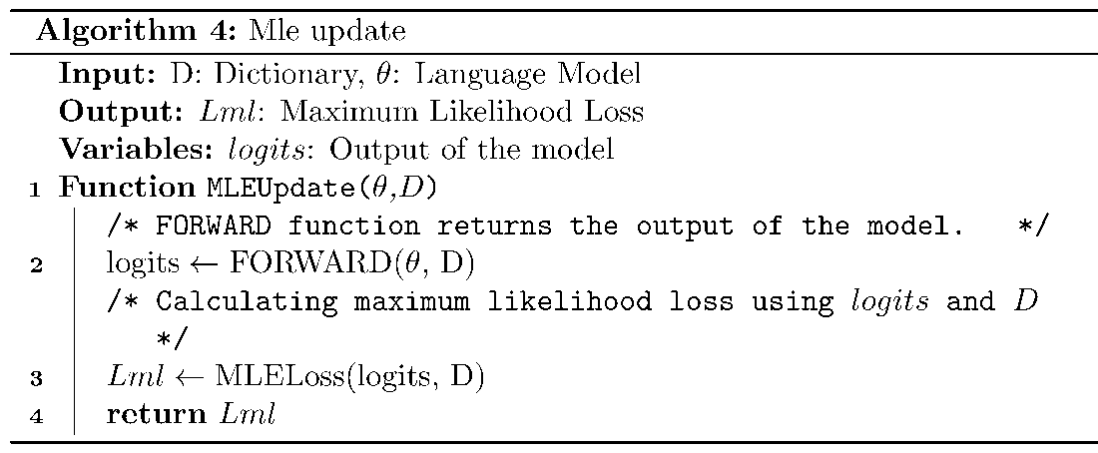


However, the results obtained by minimizing *Lml* are not always optimal. There are 2 main reasons for the degradation of results. The first is called “exposure bias” [36], which occurs when the model expects gold-standard data at each step of training, but does not receive appropriate supervision during testing, resulting in an accumulation of errors during prediction. The second is called “representation collapse” [37], which is a degradation of the pretrained language model representations during fine-tuning. Ranzato et al [36] avoided the problem of exposure bias by directly optimizing the specific discrete metric instead of minimizing the *Lml* with the help of an RL-based algorithm called REINFORCE [38]. A variant of REINFORCE [38] called Self-Critical Sequence Training [39] was used in this study to directly optimize certain rewards specifically designed for TS; more information on this is provided in the following subsection.

#### Self-critical Sequence Training

**Figure 2:**
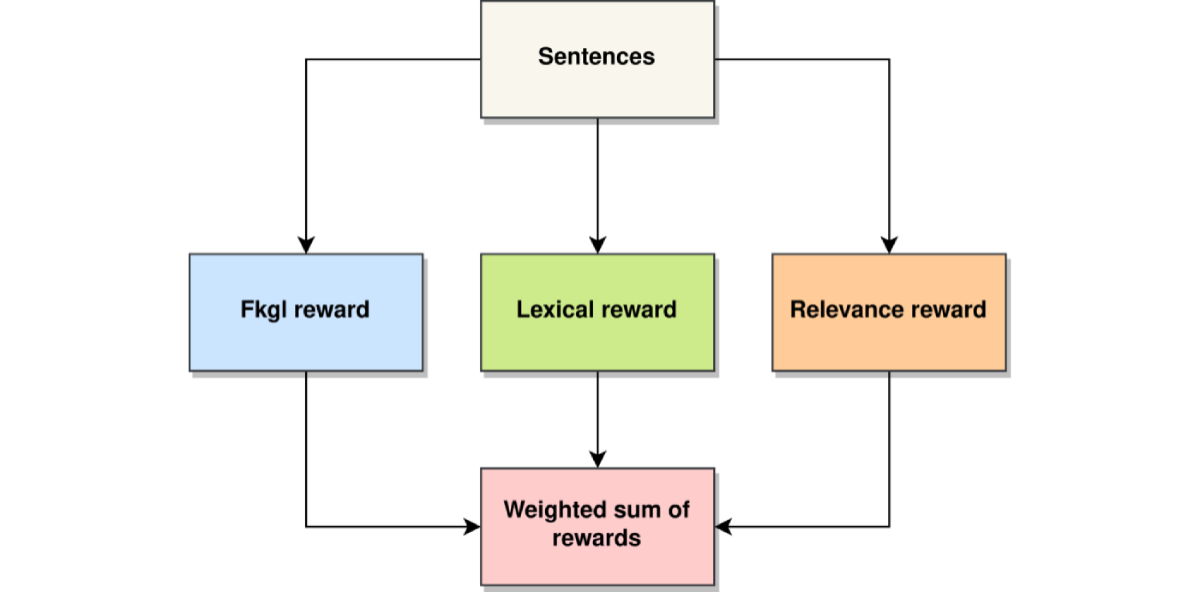
Compute Rewards function calculates a weighted sum of three rewards: Fkgl Reward, Lexical Simplicity Reward, Relevance Reward.


TS can be formulated as an RL problem, where the “agent” (language model) interacts with the environment to take “action” (next word prediction) based on a learned “policy” (*p_θ_*) defined by model parameters *θ*while observing some rewards (*R*). In this work, BART [14] was used as the language model, and the REINFORCE [38] algorithm was used to learn an optimal policy that maximizes rewards. Specifically, REINFORCE was used with a baseline to stabilize the training procedure using an objective function (*Lpg*) with a baseline reward *b* (equation 3):



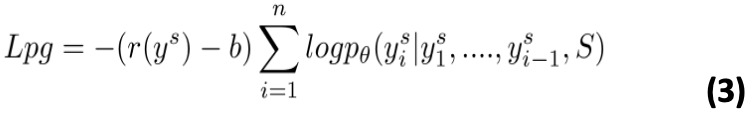



where *p_θ_*(*y_i_^s^|*...) denotes the probability of the *i*th word conditioned on a previously generated sampled sequence by the model; *r*(*y^s^*) denotes the reward computed for a sentence generated using sampling; denotes the source sentence, and *n* is the length of the generated sentence. Rewards are computed as a weighted sum of the relevance reward (*R_cosine_*), *R_Flesch_*, and lexical simplicity reward (*R_lexical_*; Figure 2) and are given by:



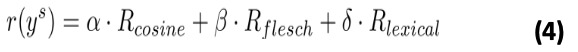



where *α*, *β*, and *d* are the weights associated with the rewards, respectively.

To approximate the baseline reward, Self-Critical Sequence Training [39] was used. The baseline was calculated by computing reward values for a sentence that has been generated using greedy decoding *r*(y*) by the current model and its computation is described in Algorithm 5. The loss function is defined in equation 5:



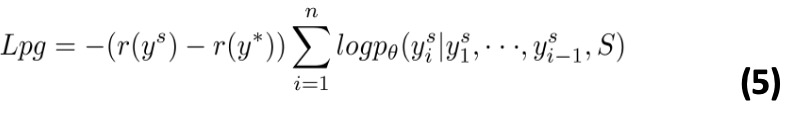



where y* denotes the sentence generated using greedy decoding. More details on greedy decoding are described in Multimedia Appendix 1 (see also [8,14,17,25,26,39-42]).

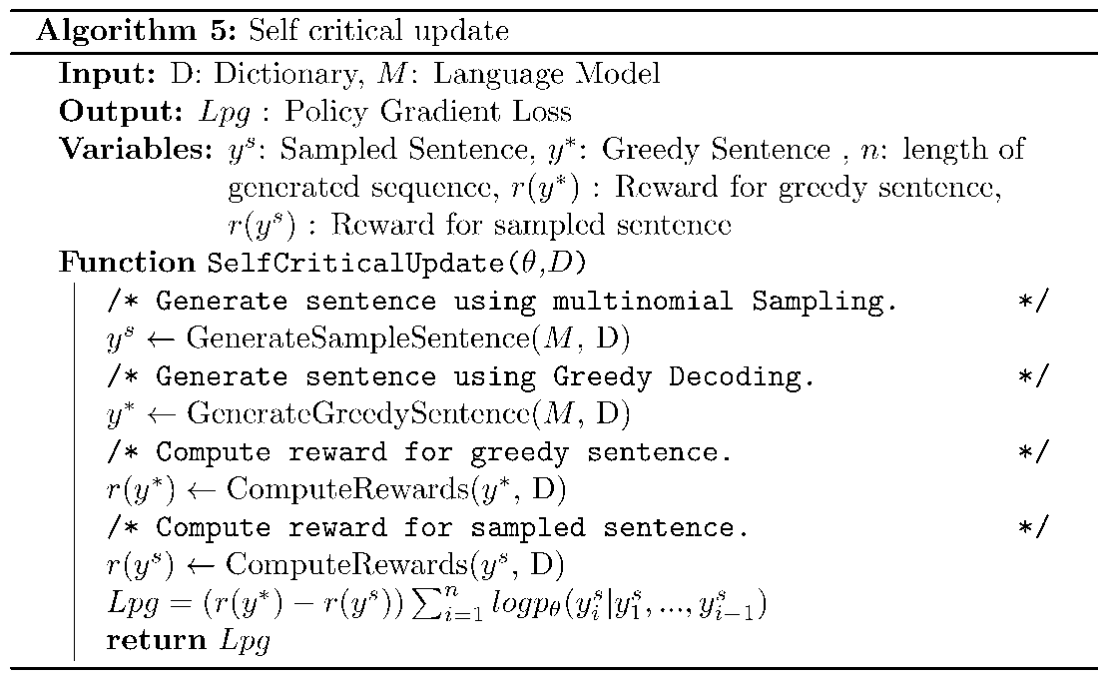


Intuitively, by minimizing the loss described in equation 5, the likelihood of choosing the samples sequence (*y^s^*) is promoted if the reward obtained for sampled sequence, *r*(*y^s^*), is greater than the reward obtained for the baseline rewards, that is, the samples that return higher reward than *r*(y*). The samples that obtain a lower reward are subsequently suppressed. The model is trained using a combination of *Lml* and policy gradient loss similar to [43]. The overall loss is given as follows:

*L* = *γLpg* + (1 – *γ*)*Lml*
** (6)**

where *γ* is a scaling factor that can be tuned.

### Summary of the Training Process

**Figure 3:**
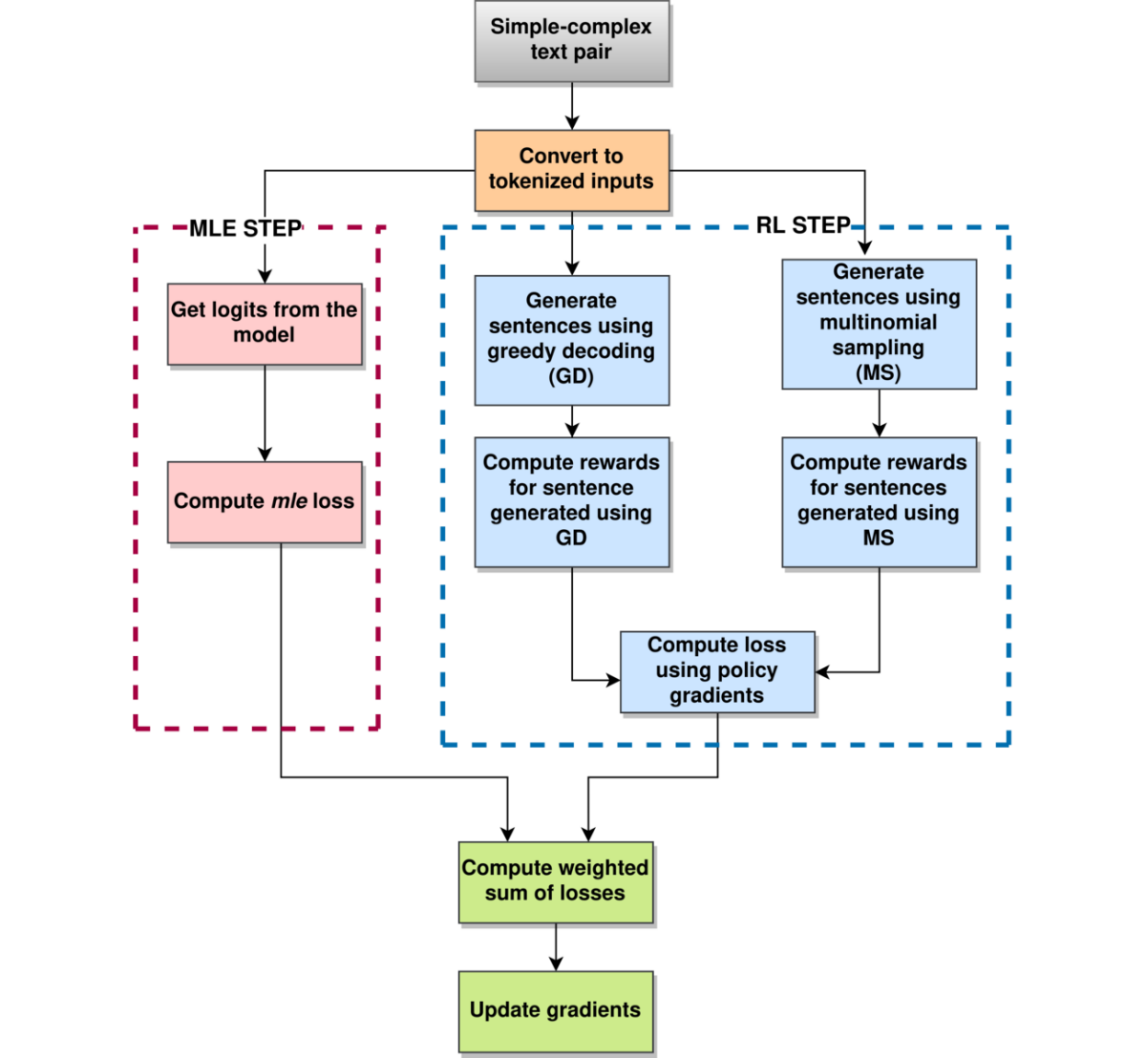
Reinforcement learning–based training procedure for TESLEA. MLE: maximum likelihood estimation; RL: reinforcement learning.

Overall, the training procedure follows a 2-step approach. As the pretrained BART [14] was not trained on the medical domain–related text, it was first fine-tuned on the document-level paired data set [8] by minimizing the *Lml* (maximum likelihood estimation [MLE]; equation 2). In the second part, the fine-tuned BART model was trained further using RL. The RL procedure of TESLEA involves 2 steps: (1) the RL step and (2) the MLE optimization step, which are both shown in Figure 3 and further described in Algorithm 6. The given simple-complex text pairs are converted to tokens as required by the BART model. In the MLE step, these tokens are used to compute *logits* from the model, and then finally MLE loss is computed. In the RL step, the model generates simplified text using 2 decoding strategies: (1) greedy decoding and (2) multinomial sampling. Rewards are computed as weighted sums (Figure 3) for sentences generated using both the decoding strategies. These rewards are then used to calculate the loss for the RL step. Finally, a weighted sum of losses is computed that is used to estimate the gradients and update model parameters. All the hyperparameter settings used are included in Multimedia Appendix 2 (see also [8,12,29,33,34,44-47]).


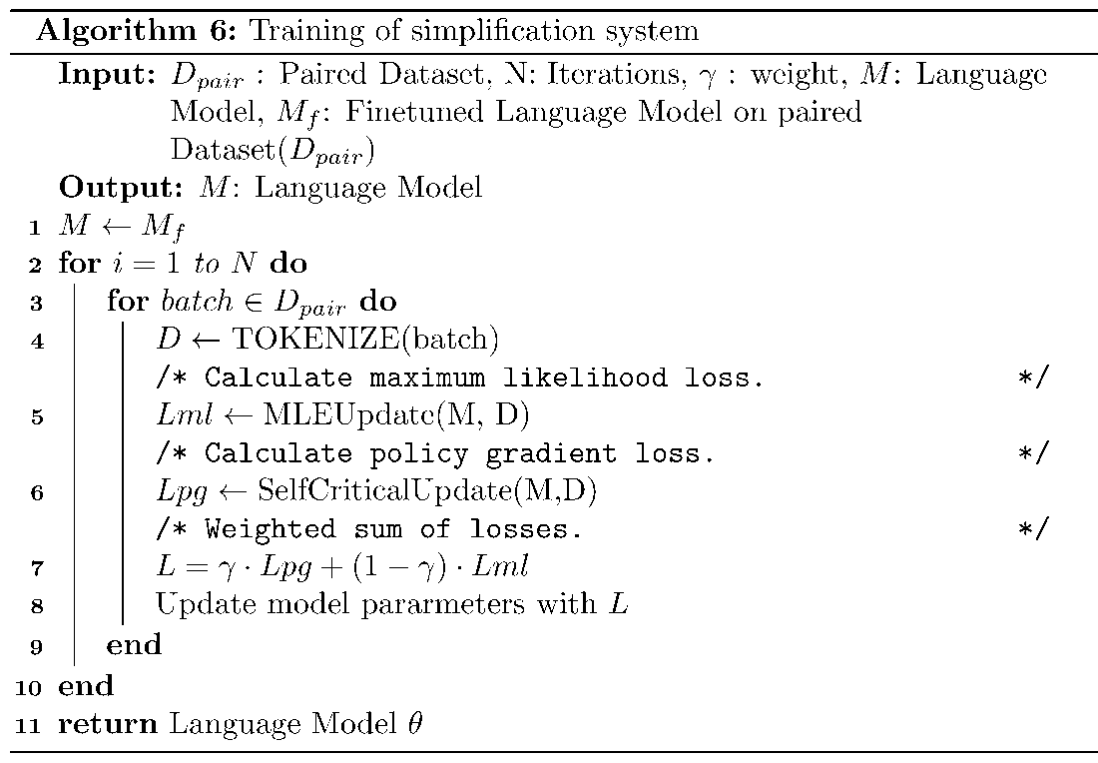


### Automatic Metrics

Two readability indices were used to perform automatic evaluations of the generated text, namely, FKGL and Automatic Readability Indices (ARIs). The SARI score is a standard metric for TS. The F-1 versions of ROUGE-1 and ROUGE-2 [44] scores were also reported. Readers can find more details about these metrics in Multimedia Appendix 2. To measure the quality of the generated text, the criteria proposed by Yuan et al [45] were used, which are mentioned in the “Automatic Evaluation Metrics” section in Multimedia Appendix 2. The criteria proposed by Yuan et al [45] can be automatically computed using a language model–based metric called “BARTScore.” Further details on how to use BARTScore to measure the quality of the generated text are also mentioned in Multimedia Appendix 2.

### Human Evaluations

**Figure 4:**
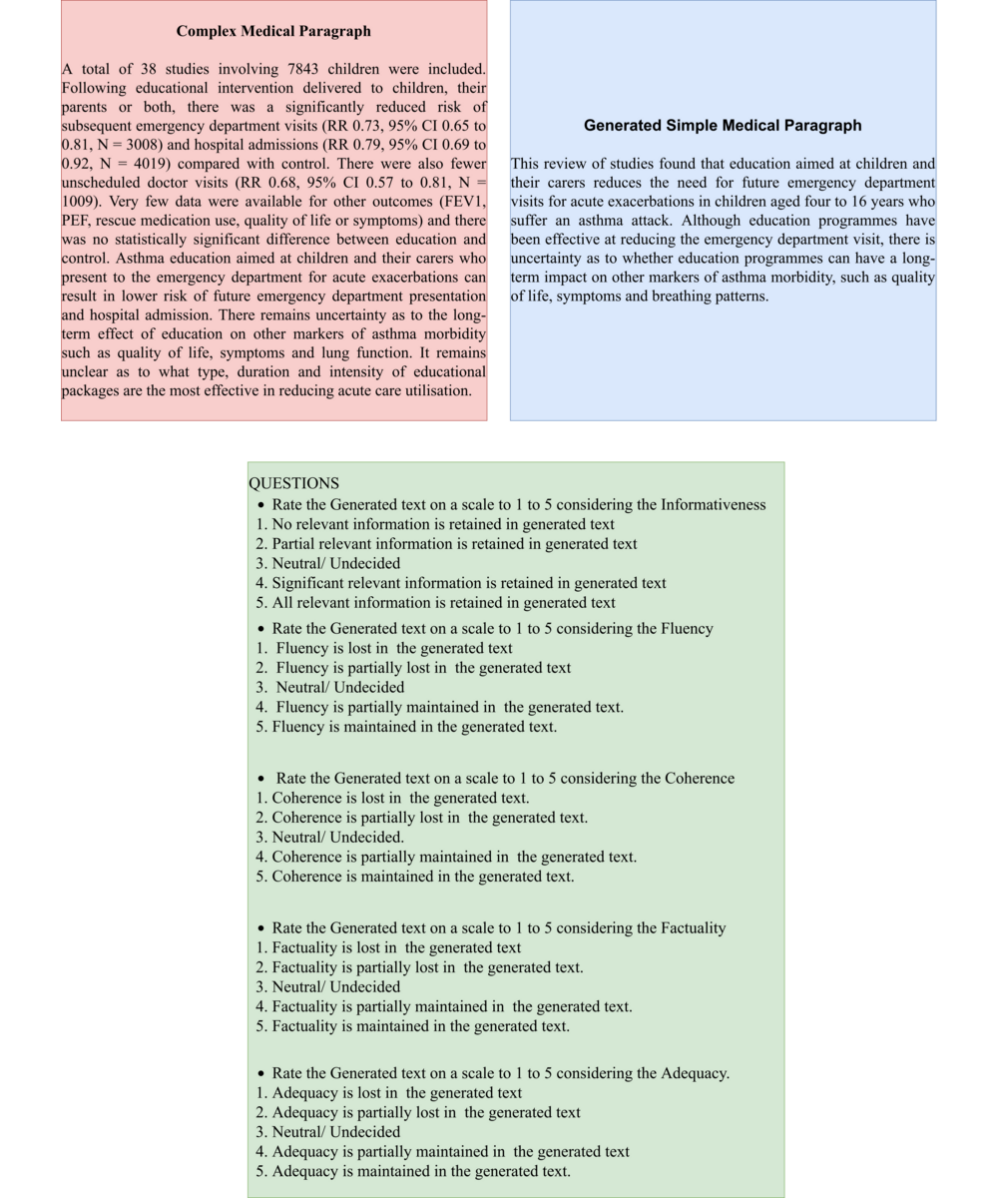
A sample question seen by the human annotator.

In this study, 3-domain experts judge the quality of the generated text based on the factors mentioned in the previous section. The evaluators rate the text on a Likert scale from 1 to 5. First, simplified test data were generated using TESLEA, and then 51 generated paragraphs were randomly selected, creating 3 subsets containing 17 paragraphs each. Every evaluator was presented with 2 subsets, that is, a total of 34 complex-simple TESLEA-generated paragraphs. The evaluations were conducted via Google Forms, and the human annotators were asked to measure the quality of simplification for informativeness (INFO), fluency (FLU), coherence (COH), factuality (FAC), and adequacy (ADE) (Figure 4). All the data collected were stored in CSV files for statistical analysis.

## Results

### Overview

This section consists of 3 subsections, namely, (1) Baseline Models, (2) Automatic Evaluations, and (3) Human Evaluations. The first section highlights the baseline models used for comparison and analysis. The second section discusses the results obtained by performing automatic evaluations of the model. The third and final section discusses the results obtained from human assessments and analyzes the relationship between human annotations and automatic metrics.

### Baseline Models

TESLEA is compared with other strong baseline models and their details are discussed below:

- BART-Fine-tuned: BART-Fine-tuned is a BART-large model fine-tuned using an *Lml* on the data set proposed by Devaraj et al [8]. Studies have shown that large pretrained models often perform competitively when fine-tuned for downstream tasks, thus making this a strong competitor.
- BART-UL: Devaraj et al [8] also proposed BART-UL for paragraph-level medical TS. It is the first model to perform paragraph-level medical TS and has achieved strong results on automated metrics. BART-UL was trained using an unlikelihood objective function that penalizes the model for generating technical words (ie, complex words). Further details on the training procedure of BART-UL are described in Multimedia Appendix 1.
- MUSS: MUSS [17] is a BART-based language model that was trained by mining paraphrases from the CCNet corpus [18]. MUSS was trained on a data set consisting of 1 million paraphrases, helping it achieve a strong SARI score. Although MUSS is trained on a sentence-level data set, it still serves as a strong baseline for comparison. Further details on the training procedure for MUSS are discussed in Multimedia Appendix 1
  .
- Keep it Simple (KIS): Laban et al [26] proposed an unsupervised approach for paragraph-level TS. KIS is trained using RL and uses the GPT-2 model as a backbone. KIS has shown strong performance on SARI scores beating many supervised and unsupervised TS approaches. Additional details on the training procedure for KIS are described in Multimedia Appendix 1.
- PEGASUS models: PEGASUS is a transformer-based encoder-decoder model that has achieved state-of-the-art results on many text-summarization data sets. It was specifically designed for the task of text summarization. In our analysis, we used 2 variants of PEGASUS models, namely, (1) PEGASUS-large, the large variant of Pegasus model, and (2) PEGASUS-pubmed-large, the large variant of the PEGASUS model that was pretrained on a PubMed data set. Both the PEGASUS models were fine-tuned using *Lml* on the data set proposed by Devaraj et al [8]. For more information regarding the PEGASUS model, the readers are suggested to refer to [46].

The models described above are the only ones available for medical TS as of June 2022.

### Results of Automatic Metrics

The metrics used for automatic evaluation are FKGL, ARI, ROUGE-1, ROUGE-2, SARI, and BARTScore. The mean readability indices scores (ie, FKGL and ARI) obtained by various models are reported in Table 1. ROUGE-1, ROUGE-2, and SARI scores are reported in Table 2 and BARTScore is reported in Table 3.

**Table 1:** Flesch-Kincaid Grade Level and Automatic Readability Index for the generated text.^a^

Text			Flesch-Kincaid Grade Level	Automatic Readability Index
**Baseline**				
	Technical abstracts	14.42		15.58
	Gold-standard references	13.11		15.08
**Model generated**				
	BART-Fine-tuned	13.45		15.32
	BART-UL	11.97		13.73^b^
	TESLEA	11.84^b^		13.82
	MUSS^c^	14.29		17.29
	Keep it Simple	14.15		17.05
	PEGASUS-large	14.53		17.55
	PEGASUS-pubmed-large	16.35		19.8

^a^TESLEA significantly reduces FKGL and ARI scores when compared with plain language summaries.

^b^Best score.

^c^MUSS: multilingual unsupervised sentence simplification.

**Table 2:** ROUGE-1, ROUGE-2, and SARI scores for the generated text.^a^

Model	ROUGE-1	ROUGE-2	SARI
BART-Fine-tuned	0.40	0.11	0.39
BART-UL	0.38	0.14	0.40^b^
TESLEA	0.39	0.11	0.40^b^
MUSS^c^	0.23	0.03	0.34
Keep it Simple	0.23	0.03	0.32
PEGASUS-large	0.44^b^	0.18^b^	0.40^b^
PEGASUS-pubmed-large	0.42	0.16	0.40^b^

^a^TESLEA achieves similar performance to other models. Higher scores of ROUGE-1, ROUGE-2, and SARI are desirable.

^b^Best performance.

^c^MUSS: multilingual unsupervised sentence simplification.

**Table 3:** Faithfulness Score and F-score for the generated text by the models.^a^

Models	Faithfulness Score	F-score
BART-Fine-tuned	0.137	0.078
BART-UL	0.242	0.061
TESLEA	0.366^b^	0.097^b^
MUSS^c^	0.031	0.029
Keep it Simple	0.030	0.028
PEGASUS-large	0.197	0.073
PEGASUS-pubmed-large	0.29	0.063

^a^Higher scores of Faithfulness and F-score are desirable.

^b^Highest score.

^c^MUSS: multilingual unsupervised sentence simplification.

#### Readability Indices, ROUGE, and SARI Scores

The readability indices scores reported in Table 1 suggest that the FKGL scores obtained by TESLEA are better (ie, a lower score) when compared with the FKGL scores obtained by comparing technical abstracts (ie, complex medical paragraphs available in the data set) with the gold-standard references (ie, simple medical paragraphs corresponding to the complex medical paragraphs). Moreover, TESLEA achieves the lowest FKGL score (11.84) when compared with baseline models, indicating significant improvement in the TS. The results suggest that (1) BART-based transformer models are capable of performing simplification at the paragraph level such that the outputs are at a reduced reading level (FKGL) when compared with technical abstracts, gold-standard references, and baseline models. (2) The proposed method to optimize TS-specific rewards allows the generation of text with greater readability than even the gold-standard references, as indicated by the FKGL scores in Table 1. The reduction in FKGL scores can be explained by the fact that FKGL was a part of a reward (*R_Flesch_*) that was directly being optimized.

In addition, we report the SARI [12] and ROUGE scores [44] as shown in Table 2. SARI is a standard automatic metric used in sentence-level TS tasks. The ROUGE score is another standard metric in text summarization tasks. The results show that TESLEA matches the performance of baseline models on both ROUGE and SARI scores. Although there are no clear patterns when ROUGE and SARI scores are considered, there are differences in the quality of text generated by these models and these are explained in the “Text Quality Measure” subsection.

#### Text Quality Measure

There has been significant progress in designing automatic metrics that are able to capture linguistic quality of the text generated by language models. One such metric that is able to measure the quality of generated text is BARTScore [45]. BARTScore has shown strong correlation with human assessments on various tasks ranging from machine translation to text summarization. BARTScore has 4 different metrics (ie, Faithfulness Score, Precision, Recall, F-score), which can be used to measure different qualities of generated text. Further details on how to use BARTScore are mentioned in Multimedia Appendix 2.

According to the analysis conducted by Yuan et al [45], Faithfulness Score measures 3 aspects of generated text via COH, FLU, and FAC. The F-score measures 2 aspects of generated text (INFO and ADE). In our analysis, we use these 2 variants of BARTScore to measure COH, FLU, FAC, INFO, and ADE. TESLEA achieves the highest values (Table 3) of Faithfulness Score (0.366) and F-score (0.097), indicating that the rewards designed for the purpose of TS not only help the model in generating simplified text but also on some level preserve the quality of generated text. The F-scores of all the models are relatively poor (ie, scores closer to 1 are desirable). One of the reasons for low F-scores could be the introduction of misinformation or hallucinations in the generated text, a common problem for language models, which could be addressed by adapting training strategies that focus on INFO via the help of rewards or objective functions.

For qualitative analysis we randomly selected 50 sentences from the test data and calculated the average number of tokens based on BART model vocabulary. For the readability measure, we calculated the FKGL scores of these generated texts and noted any textual inconsistencies such as misinformation. The analysis revealed that the text generated by most models was significantly smaller than the gold-standard references (Table 4). Furthermore, TESLEA- and BART-UL–generated texts were significantly shorter compared with other baseline models and TESLEA had the lowest FKGL score among all the models as depicted in Table 4.

**Figure 5:**
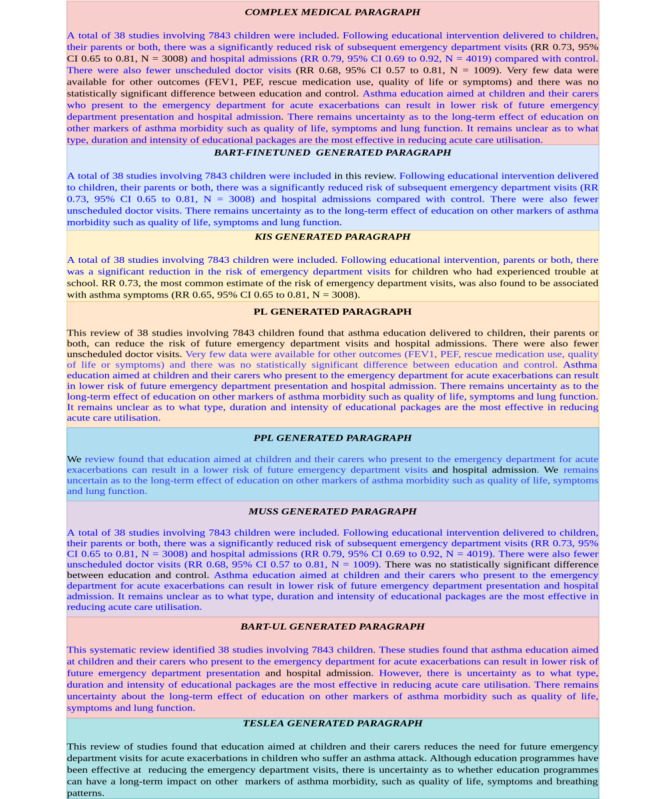
Comparison of Text Generated by all the models. The highlighted blue text indicates copying. CI: Confidence Interval; FEV: Force Expiratory Volume; N: Population size; PEV: Peak Expiratory Flow; RR: Respiratory Rate.

From a qualitative point of view, the sentences generated by most baseline models involve significant duplication of text from the original complex medical paragraph. The outputs generated by the KIS model were incomplete and appear “noisy” in nature. One of the reasons for the noise generation could be because of unstable training due to lack of a huge corpus of domain-specific data. BART-UL–generated paragraphs are simplified as indicated by the FKGL and ARI scores, but they are extractive in nature (ie, the model learns to select simplified sentences from the original medical paragraph and combines them to form a simplification). PEGASUS-pubmed-​large–generated paragraphs are also extractive in nature and similar to BART-UL–generated paragraphs, but it was observed that they were grammatically inconsistent. In contrast to baseline models, the text generated by TESLEA was concise, semantically relevant, and simple, without involving any medical domain–related complex vocabulary. Figure 5 shows an example of text generated by all the models, with blue text indicating the copied text.

**Figure 6:**
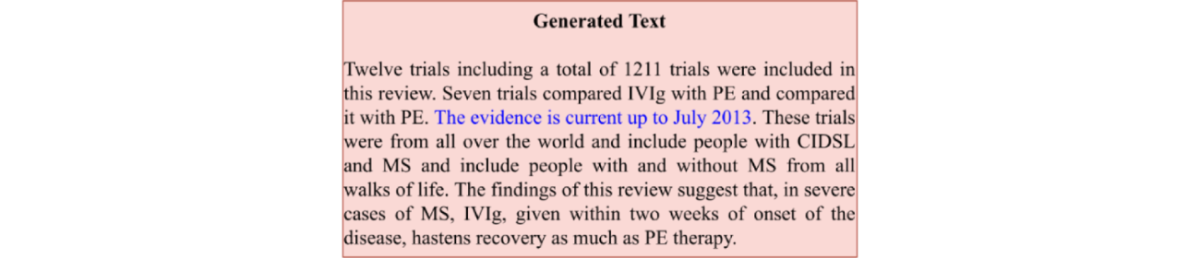
Example of misinformation found in Generated text. CIDSL: Cornelia de Lange syndrome; IVIg: Intravenous immune globulin; MS: Multiple Sclerosis; PE: plasma exchange.

In addition to the duplicated text, the models also induced misinformation in the generated text. The most common form of induced misinformation observed was “The evidence is current up to [date],” as shown in Figure 6. This text error occurred due to the structure of the data (ie, PLS contains statements related to this research, but these statements were not in the original text; thus, the model attempted to add these statements to the generated text although it is not factually correct). Thus considerable attention should be paid to including FAC measures in the training regime of these models. For a more complete assessment of the quality of simplification, human evaluation was conducted using domain experts for the text generated by TESLEA.

**Table 4:** Average number of tokens and average Flesch-Kincaid Grade Level scores for selected samples.

Model	Number of tokens	Flesch-Kincaid Grade Level
Technical abstracts	498.11	14.37
Gold-standard references	269.74	12.77
TESLEA	131.37	12.34
BART-UL	145.08	12.66
Keep it Simple	187.59	13.78
Multilingual unsupervised sentence simplification	193.07	13.86
PEGASUS-large	272.04	13.93
PEGASUS-pubmed-large	150.00	15.09

### Human Evaluations

For this research, 3 domain experts assessed the quality of generated text, based on factors of INFO, FLU, COH, FAC, and ADE, as proposed by Yuan et al [45], which are discussed in Multimedia Appendix 2. To measure interrater reliability, the percentage agreement between the annotators is calculated, and the results are shown in Table 5. The average percentage agreement for the factors of FLU, COH, FAC, and ADE is the highest, indicating that annotators agree among their evaluations.

The average Likert score for each factor is also reported by each rater (Table 6). From the data mentioned in Table 6, the raters think that the COH and FLU have the highest quality, with the ADE, FAC, and INFO also rated reasonably high.

**Figure 7:**
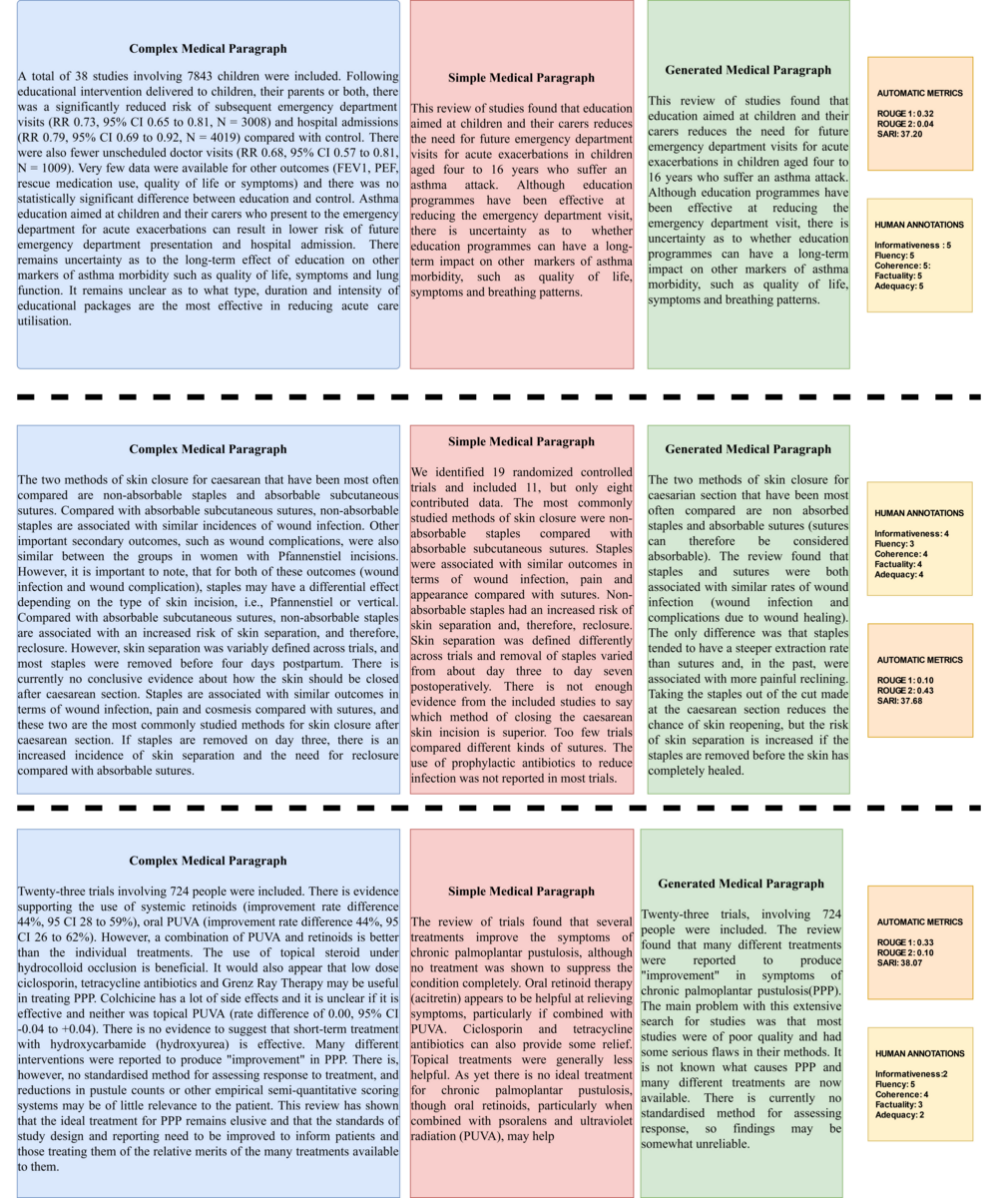
Samples of Complex, Simple (Gold) and generated medical paragraphs along with automated metrics and Human annotations.

To further assess whether results obtained by automated metrics truly signify an improvement in the quality of generated text by TESLEA, the Spearman rank correlation coefficient was calculated between human ratings and the automatic metrics for all 51 generated paragraphs (text), with the results shown in Table 7. The BARTScore has the highest correlation with human ratings for FLU, FAC, COH, and ADE compared with other metrics. A few text samples along with their human annotations and automated metric scores are shown in Multimedia Appendix 3 and Figure 7.

**Table 5:** Average percentage interrater agreement.

Interrater agreement	Informativeness, %	Fluency, %	Factuality, %	Coherence, %	Adequacy, %
A1^a^ and A2^b^	82.35	82.35	82.35	70.59	82.35
A1 and A3^c^	70.59	58.82	70.59	70.59	70.59
A3 and A2	52.94	70.59	74.51	74.51	64.71
Average (% agreement)	68.63	70.59	74.51	74.51	72.55

^a^A1: annotator 1.

^b^A2: annotator 2.

^c^A3: annotator 3.

**Table 6:** Average Likert score by each rater for informativeness, fluency, factuality, coherence, and adequacy.

Rater	Informativeness	Fluency	Factuality	Coherence	Adequacy
A1	3.82	4.12	3.91	3.97	3.76
A2	3.50	4.97	3.59	4.82	3.68
A3	4.06	3.94	3.85	3.94	3.85
Average Likert score	3.79	4.34	3.78	4.24	3.76

**Table 7:** Spearman rank correlation coefficient between automatic metrics and human ratings for the text generated by TESLEA.

Metric	Informativeness	Fluency	Factuality	Coherence	Adequacy
ROUGE-1	0.18^a^	–0.04	–0.01	–0.05	0.06
ROUGE-2	0.08	–0.01	–0.05	–0.04	0.05
SARI	0.09	–0.66	–0.13	–0.01	0.01
BARTScore	0.08	0.32^a^	0.38^a^	0.22^a^	0.07^a^

^a^Best result.

## Discussion

### Principal Findings

The most up-to-date research about biomedicine is often inaccessible to the general public due to the domain-specific medical terminology. A way to address this problem is by creating a system that converts complex medical information into a simpler form, thus making it accessible to everyone. In this study, a TS approach was developed that can automatically simplify complex medical paragraphs while maintaining the quality of the generated text. The proposed approach trains the transformer-based BART model to optimize rewards specific for TS, resulting in increased simplicity. The BART model is trained using the proposed RL method to optimize certain rewards that help generate simpler text while maintaining the quality of generated text. As a result, the trained model generates simplified text that reduces the complexity of the original text by 2-grade points, when measured using the FKGL [29]. From the results obtained, it can be concluded that TESLEA is effective in generating simpler text compared with technical abstracts, the gold-standard references (ie, simple medical paragraphs corresponding to complex medical paragraphs), and the baseline models. Although previous work [8] developed baseline models for this task, to the best of our knowledge, this is the first time RL is being applied to the field of medical TS. Moreover, previous studies failed to analyze the quality of the generated text, which this study measures via the factors of FLU, FAC, COH, ADE, and INFO. Manual evaluations of TESLEA-generated text were conducted with the help of domain experts using the aforesaid factors and further research was conducted to analyze which automatic metrics agree with manual annotations using the Spearman rank correlation coefficient. The analysis revealed that BARTScore [45] best correlates with the human annotations when evaluated for a text generated by TESLEA, indicating that TESLEA learns to generate semantically relevant and fluent text, which conveys the essential information mentioned in the complex medical paragraph. These results suggest that (1) TESLEA can perform TS of medical paragraphs such that outputs are simple and maintain the quality, (2) the rewards optimized by TESLEA help the model capture syntactic and semantic information, increasing the FLU and COH of outputs, as witnessed when the outputs are evaluated by BARTScore and human annotators.

### Limitations and Future Work

Although this research is a significant contribution to the literature on medical TS, the proposed approach does have a few limitations, addressing which can result in even better outputs. TESLEA can generate simpler versions of the text, but in some instances, it induces misinformation, resulting in reduced FAC and INFO of the generated text. Therefore, there is a need to design rewards that consider the FAC and INFO of the generated text. We also plan to conduct extensive human evaluations on a large scale for the text generated by various models (eg, KIS, BART-UL) using domain experts (ie, physicians and medical students).

Transformer-based language models are sensitive to the pretraining regime, so a possible next step is to pretrain a language model on domain-specific raw data sets such as PubMed [40], which will help develop domain-specific vocabulary for the model. Including these strategies may help in increasing the simplicity of the generated text.

### Conclusion

The interest in and need for TS in the medical domain are of growing interest as the quantity of data is continuously increasing. Automated systems, such as the one proposed in this paper, can dramatically increase accessibility to information for the general public. This work not only provides a technical solution for automated TS, but also lays out and addresses the challenges of evaluating the outputs of such systems, which can be highly subjective. It is the authors’ sincere hope that this work allows other researchers to build on and improve the quality of similar effort.

## Appendix

Multimedia Appendix 1 Training Procedures and Decoding Methods.

Multimedia Appendix 2 Hyperparameters and Evaluation Metrics.

Multimedia Appendix 3 Abbreviations and Examples.

## Abbreviations

ARI: Automated Readability Index
BERT: bidirectional encoder representations from transformers
FKGL: Flesch-Kincaid Grade Level
GPT: generative pretraining transformer
MLE: maximum likelihood estimation
KIS: Keep it Simple
Lml: maximum likelihood loss
LS: lexical simplification
LSTM: long short-term memory
MUSS: multilingual unsupervised sentence simplification
PLS: plain language summary
RFlesch: FKGL reward
RL: reinforcement learning
