# Supplementary material for: Medical Text Simplification Using Reinforcement Learning (TESLEA): Deep Learning–Based Text Simplification Approach
Source: JMIR Med Inform. 2022 Nov 18;10(11):e38095. doi: 10.2196/38095 (PMC9719064; doi:10.2196/38095)
Supplement: Multimedia Appendix 1 [file medinform_v10i11e38095_app1.docx]

**Training Procedures and Decoding Methods**

**Training Procedures**

**BART-UL**

Devaraj et al [8] proposed BART-UL, a model which uses the BART model as the backbone for paragraph-level medical text simplification. To ensure that the model does not generate technical words, Devaraj et al [8] adapted the concept of unlikelihood training to penalize the model whenever it generated technical words. The proposed unlikelihood training objective (ie, unlikelihood loss (UL)) [45] is calculated as follows and is shown in Equation (9) [8]


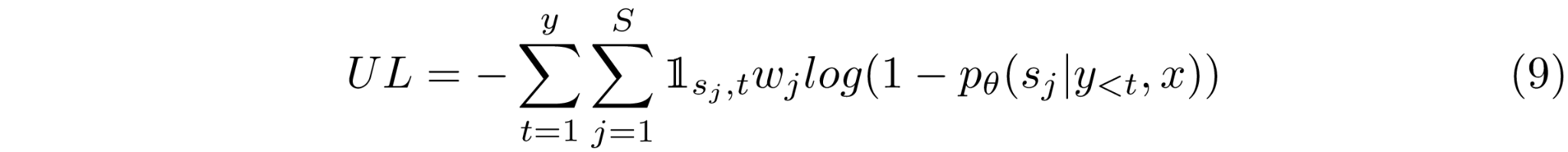


where $S$ is a set of candidate tokens, $x$ is the complex medical paragraph as the input, $y_{<t}$ is the prefix of simple medical paragraph ***y,***and $p_{\theta}(s_{j}|y_{t},x)$ is the probability assigned to token $s_{j}$ in the distribution output by BART with model parameters $\theta$ at time $t$[8]**.** The set of candidate tokens ($S$) is calculated by collecting tokens with negative weights using a bag of word logistic regression which is trained to classify whether the given paragraph is simple or complex. The unlikelihood loss mentioned in equation (9) is only applied for a given token $s_{j}$ with learned logistic regression weight $w_{j}$ if the output probability distribution of the BART model for the token indicates that it should be in the generated output. The final loss function for training BART-UL is the weighted sum of unlikelihood loss and the standard maximum likelihood loss. The proposed training method by Devaraj et al [8] helped them perform paragraph level simplification on medical text data.

**MUSS: Multilingual Unsupervised Sentence Simplification by Mining Paraphrases**

Martin et. al. [17] recently introduced MUSS, a BART [14] based language model which achieved state of the art results on TS benchmarks. MUSS was trained on a data set created by mining paraphrases from CCNET corpus. This data set is a sentence level data set ie, data set contains complex sentences and corresponding simple sentences. During the training time, MUSS uses control tokens which tell the model about important properties of target sentence. The control tokens used by MUSS are character length ratio (NumChar), replace-only Levenshtein similarity (LevSim), Word frequency ratio (WordFreq), Dependency Tree Depth Ratio (DepTreeDepth). These control tokens were first proposed by Martin et. al. [48] and their importance is given below:

- Character Length Ratio: This control token measures compression and content deletion between source and target sentence. [48]
- Levenshtein Similarity: Levenshtein similarity measures the amount of modifications done on source sentences via deletion, addition or replacement. In case MUSS they have only considered replacement as paraphrases often do not involve heavy deletion or addition operations. [48]
- Word Frequency Ratio: Word Frequency are shown to be good indicators of word complexity and hence word frequency ratio between source and target sentences serves as a proxy to measure lexical similarity. [48]
- Dependency Tree Depth Ratio (DepTreeDepth): Dependency Tree Depth Ratio is measured as maximum depth of dependency tree of the source sentence divided by that of the target sentence. DepTreeDepth serves as a proxy to syntactic simplicity. [48]

These four tokens are prepended to every source sentence while training the MUSS model. Overall, MUSS is trained on a data set consisting of one million paraphrases. In our experiments we did not further fine-tune the MUSS model as the control tokens designed are for sentence level simplification tasks whereas the data set proposed by Devaraj et al [8] is a paragraph level text simplification data set and designing the oracle tokens for a paragraph level task is out of the scope for the current paper. Although MUSS is trained on sentence level data, it still serves as a strong baseline because of the huge corpus data that was used to train the model. Due to this reason, we have included the MUSS model as a baseline.

**Keep it Simple: Unsupervised Simplification of Multi-Paragraph Text**

Laban et al [26] proposed Keep it Simple (KIS) an unsupervised reinforcement learning based approach to simplify paragraph level text. They propose a variant of SCST [40] called K-SCST in which instead of proposing one candidate simplification, the model proposes multiple candidate simplifications, computes the reward for candidates and encourages simplification which outperforms the mean reward. More formally in K-SCST, k sampled sentences are generated and rewards are computed for each candidate $R^{S1},..., R^{Sk}$ and the baseline is approximated as the mean of these rewards

($R^{S}$). The loss function is defined as follows and shown in Equation (10) [25]


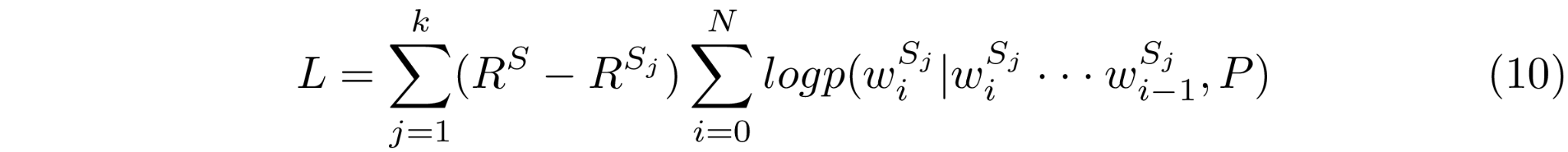


Where P is the input sentence and L is the resulting loss function and k is the number of sampled sentences. The rewards used by KIS are Salience Rewards, Lexical Simplicity Rewards, Syntactic Simplicity Rewards, and Language model-based Fluency Reward. All the rewards are unsupervised ie, they do not require any reference sentence and only require the source and generated sentences. They also have introduced guardrails to maintain length and accuracy of generated text. A GPT2 model is trained using KIS procedure on an unreleased data set of 7 million news articles. For our experiments, we fine-tuned the GPT2 model using KIS procedure on the data set proposed by Devaraj et al [8]. Since KIS is an unsupervised TS approach, it requires a lot of data to reach an optimal score. Unfortunately, the data set released by Devaraj et al [8] has only 3568 training instances and hence is not enough to ensure that model can be trained properly. We believe that having a large corpus of paragraph level medical text can help in stabilizing the training procedure. For more details about the rewards, data sets used in KIS procedure readers are suggested to refer to [26].

**Decoding Strategies**

Greedy Decoding

The transformer based language models which are used in sequence to sequence tasks usually follow an encoder-decoder structure. The encoder side takes an input sequence $(x_{1},...x_{n})$and outputs a continuous sequence of representations $(z_{1},..,z_{n}$). The decoder takes these continuous representations as input and outputs a generated sequence $(y_{1},...,y_{n})$. At each generation step the model is autoregressive, ie, it consumes previously generated tokens and outputs the probability scores to select next tokens. In greedy decoding, the token with maximal probability is always selected. In general, greedy decoding step at time *t* is denoted as follow


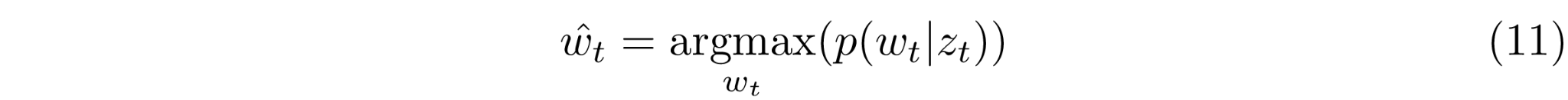


Where $\hat{w_{t}}$ denotes the next generated token, $w_{t}$ denotes previous generated token and $z_{t}$ denotes representation of input tokens obtained from encoder [49].
