# Supplementary material for: Medical Text Simplification Using Reinforcement Learning (TESLEA): Deep Learning–Based Text Simplification Approach
Source: JMIR Med Inform. 2022 Nov 18;10(11):e38095. doi: 10.2196/38095 (PMC9719064; doi:10.2196/38095)
Supplement: Multimedia Appendix 2 [file medinform_v10i11e38095_app2.docx]

**Hyperparameters and Evaluation Metrics**

**TESLEA: Hyper-Parameter Settings**

The data-split used is the same as proposed by Devaraj et al [8] with 3568 reviews in the training set, 411 in the validation set, and 480 in the test set. The pretrained BART model is initialized from a checkpoint trained on the XSum data set [34]. The model parameters are updated using AdamW [46], with a learning rate of 2e-5 for both initial fine-tuning and RL training. The model was first fine-tuned for 10 epochs and then trained for 30 epochs using RL training. From experiments performed on the validation set, we found that the optimal value for the scaling factor $\gamma$ is 0.95 and we also observed that the optimal value for weights $(\alpha, \beta, \delta)$ is 1, which leads to the best performance on the validation set as well as the test set, stabilizing the training regime. All experiments were performed on a single NVIDIA A-100 GPU with a memory size of 40GB.

We mainly experimented with 3 variants of the BART-model, namely BART-base, BART-large and BART-large-Xsum. The variants differed in the model size, pre-training data and Batch Size every other parameter was kept constant to stabilize the training regime. Table 6 describes the variants of BART model along with model size, pre-training data, batch size, Time to train (reported in days), Inference speed per sample on Test Data set (reported in seconds) and the FKGL score obtained by each model. One can observe from Table 8 that the BART-large-xsum variant performs the best on FKGL score.

| Model | Model Parameters (Millions) | Pre-Training Data | Batch Size | Time to Train (Approx. Number of Days) | Inference Speed (Seconds) | FGKL Score |
| --- | --- | --- | --- | --- | --- | --- |
| Bart-Base | 139 M | English Wikipedia + Book Corpus | 2 | 4 | 1.3 | 13.23 |
| Bart-Large | 406 M | English Wikipedia + Book Corpus | 1 | 7 | 1.75 | 13.48 |
| Bart-Large-Xsum | 406 M | English Wikipedia + Book Corpus + Xsum | 1 | 7 | 1.75 | **11.84** |

Table 8: Information about BART-variants and other parameters.

**Automatic Evaluations Metrics**

**ROUGE Scores**: Lin et al [42] proposed an automatic metric called "Recall-Oriented Understudy for Gisting Evaluation" (ROUGE) for the task of evaluating text summarization models. Rouge scores are recall based metrics and are computed by measuring n-gram overlap between generated summary and target summary. There are various variants of ROUGE scores, specifically, we use ROUGE-1 and ROUGE-2 scores because they have shown a strong correlation to human judgements for text summarization tasks [42].

**SARI Score**: Xu et al [12] proposed an automatic metric for evaluations of text simplification called "SARI" which uses F1-score of n-gram operations to measure simplicity. It computes an average of F1-scores for three n-gram operations: additions, keeps, and deletions, which are calculated based on the recall $R(n)$ and precision $P(n)$, based on the intersections of the input, output, and reference sets [35]. For each operation (ie, add, keep, and deletion) F1-score is computed and SARI is the average of all the F1-scores as shown in Equation (7) below:


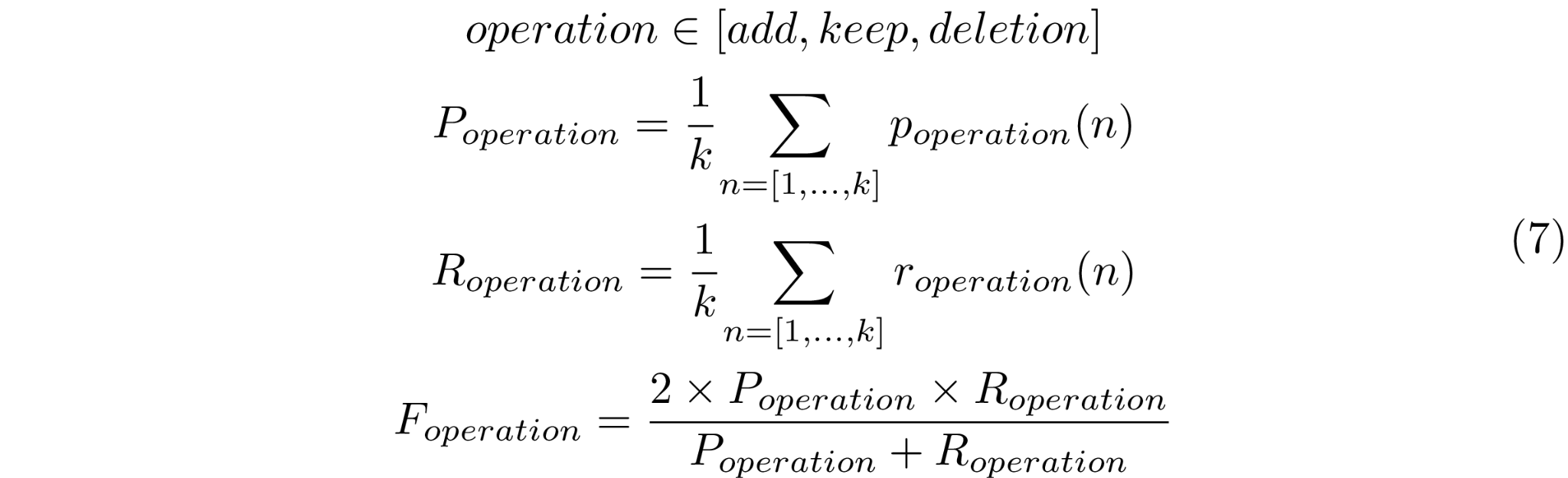


For more information regarding the SARI score, the readers are suggested to refer to [12].

**BARTScore**: Yuan, Neubig, and Liu [43] framed the problem of evaluating generated text as a text generation problem. BARTScore helps to assess the quality of the generated text. They evaluate generated text via the probability of it being generated from other text (ie, source texts or reference texts) or vice versa. The BART [14] model is used to estimate the probabilities required to calculate the given scores. Given one text *y* and another text *x*, BARTScore is calculated using weighted probability and is calculated by Equation (8) below [43]:


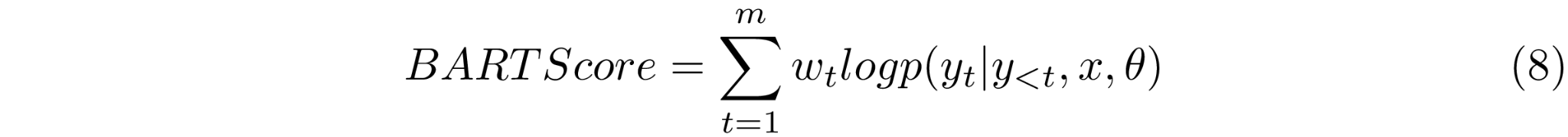


where m is the length of y; $y_{<t}$ denotes preceding tokens before the position t; $\theta$ are model parameters and $w_{t}$ are the weights associated with different tokens, however Yuan, Neubig, and Liu [44] weigh each token equally.

The criteria proposed by Yuan, Neubig, and Liu [43] to measure the quality of the generated text is given below:

- **Informativeness (INFO)**: Does the generated text capture the important ideas of the source text [44].
- **Fluency (FLU)**: Does the generated text has no formatting problems or grammatical errors that increase the difficulty to read the text. [44].
- **Coherence (COH)**: Whether the generated text relates from sentence to sentence in a logically consistent order to present information about a topic [44].
- **Factuality (FAC)**: Whether the generated text contains only statements supported by the source text (ie, no new information is being introduced) [44].
- **Adequacy (ADE)**: Whether the generated text conveys the same meaning as the source text, and none of the important information is missing or added or misreported [44].

Yuan, Neubig, and Liu [43] also introduced four different settings for the evaluation of the criteria mentioned above. For a given source text ($s$), generated text ($h$), and reference text ($r$), the settings are defined as follows:

- Faithfulness Score (s→h): This score measures how likely it is that generated text can be obtained given the source text. Faithfulness score can be used to measure factors of coherence, fluency, factuality, and relevance.
- Precision (r→h): This score measures how likely generated text can be obtained from reference texts.
- Recall (h→r): This score measures how likely generated reference texts can be obtained from generated text.
- F-score (r→h): F-score is the average of precision and recall scores and can be used to measure adequacy and informativeness.

**Flesch-Kincaid Grade Level score**: Kincaid et al [30] proposed the FKGL which gives a score that indicates a certain level that must be obtained to understand a particular text. A lower value of the FKGL score indicates that a particular text is simpler to read, and a higher score indicates that the text is complex [30].
