# Supplementary material for: Medical Text Simplification Using Reinforcement Learning (TESLEA): Deep Learning–Based Text Simplification Approach
Source: JMIR Med Inform. 2022 Nov 18;10(11):e38095. doi: 10.2196/38095 (PMC9719064; doi:10.2196/38095)
Supplement: Multimedia Appendix 3 [file medinform_v10i11e38095_app3.docx]

**Abbreviations and Examples**

| **Description** | **Abbreviation** |
| --- | --- |
| Text Simplification | TS |
| Reinforcement Learning | RL |
| Self Critical Sequence Training | SCST |
| Flesch-Kincaid Grade Level | FKGL |
| Recall-Oriented Understudy for Gisting Evaluation | ROUGE |
| Fluency | FLU |
| Coherence | COH |
| Factuality | FAC |
| Informativeness | INFO |
| Adequacy | ADE |
| Average Likert Score | ALS |

Table 9: A list of abbreviations

Code files are open-sourced and available here: <https://github.com/Atharva-Phatak/TESLEA>

**
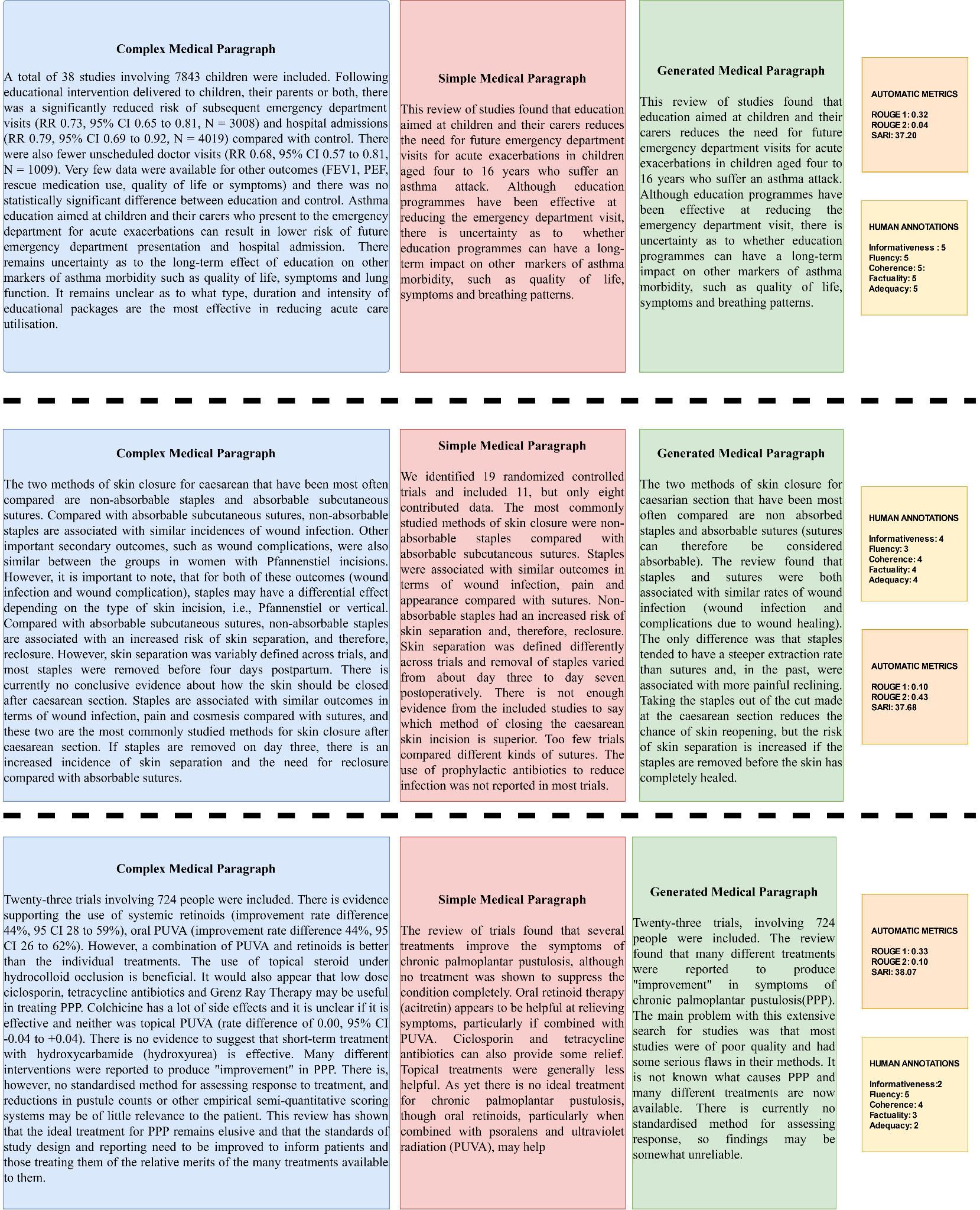
**

Figure 7: Samples of Complex, Simple (Gold) and generated medical paragraphs along with automated metrics and Human annotations.
